# Abstracts from Foreign Journals

**Published:** 1903-04

**Authors:** S. J. J. Harger

**Affiliations:** Philadelphia, Pa.


### ABSTRACTS FROM FOREIGN JOURNALS.

### FRENCH.

UNDER THE DIRECTION OF S. J. J. HARGER, V.M.D.,
PHILADELPHIA, PA.

ARTICULAR RHEUMATISM IN THE HORSE. The following case related by Altmann clearly demonstrates articular rheumatism in the horse. The subject, a two-year-old filly running in pasture during a cold and wet summer, presented the following symptoms: Pronounced emaciation, poor appetite, intense lameness in the posterior right leg; stifle hot, painful, swollen, with distended synovial membrane; temperature 39.6° C. The following day the hock became swollen, hot, and painful. Two days afterward the stifle symptoms were improved and the articulations of the phalanges in the forelegs became affected. After eight days of treatment the filly was much improved. Then the two knees became inflamed and the symptoms in the right stifle returned, the temperature varying from 39.8° to 41°, and in fifteen days the animal died.

*Autopsy. Stifle:* Abundant synovia mixed with fibrinous clots, synovial membrane congested, articular cartilage red. *Hock* and *knee:* Increased quantity of synovia, synovials red, cartilage exfoliated. *Pastern:* Vascular synovial. *Heart:* Mitral valves retracted, thickened, irregular and roughened by grayish-white productions, varying in size from that of a pinhead to that of a pea.—*Berliner Thierärz Wochen.*

COLLOIDAL SILVER AND ITS THERAPEUTIC APPLICATIONS. Colloidal silver, or collargol, has the advantage over the other silver preparations, citral and lactal, in that it is not irritating and does not coagulate albumin. It is prepared by the action of citrate of silver upon sulphate of iron. It presents itself in the form of small black grains easily broken and soluble in twenty-five parts of water.

Collargol is preferably administered by friction upon the skin (collargol 14 parts, benzoated lard 100 parts, and white wax 10 parts), or injected into the veins (1 : 100 in distilled water). In order to prevent infection after surgical operations upon the peritoneum or the uterus, Credé introduced into the peritoneal and uterine cavities from 1 to 4 pills of 0.05 gramme of collargol. Schlossmann proved that microbe cultures can with impunity be injected into the peritoneal cavity of the rabbit if a small quantity of collargol be at the same time introduced. Intravenous injections are very well supported, even in doses larger than those necessary to obtain a therapeutic result.

Therapeutically collargol was first employed in veterinary medicine. In 1898 Dickerhoff successfully treated anasarca by intravenous infections of 0.5 gramme, repeated in grave cases. In 1902 more than sixty cases were successfully treated. Kruger obtained a rapid recovery in an aggravated case of intestinal charbon by injecting 5 grammes intravenously in 250 grammes of water.

Dysentery in calves was treated with the same success. In stables where from 60 to 90 per cent. of the calves died, Evers saved all of them by injecting during the three days following calving 5 centigrammes of collargol into the jugular. In one place twenty-two so treated escaped the disease, while in a control lot not injected three died. On another farm fifteen calves injected escaped the infection, while before the use of collargol six died.

Stampff relates that on one farm where dysentery of calves was endemic for several years, occasioning a mortality of from 30 to 40 per cent., intravenous injections of collargol during the twenty-four hours after birth completely eradicated the disease. Upon seventy-nine animals treated, not one became infected, while two calves not injected died, and so did two others brought from a neighboring farm and not treated.

Colloidal silver is bactericidal, although not so in a high degree, to which Credé attributed its therapeutic action.

Netter and Salomon, on the other hand, compare its action to that of colloidal platinum, and claim that, like the latter, it has catalytic properties, acting like a ferment upon certain tissues of the organism.

Collargol is absorbed by the skin. It is eliminated by the intestinal tract. Intravenous injections rapidly disappear from the circulation. The lost traces of it can be found in the spleen, kidney, and intestine.—*Presse Médicale.*

IMMUNIZATION OF YOUNG BOVIDA AGAINST TUBERCULOSIS. Behring has demonstrated that young bovida receiving into the veins 4 milligrammes of a dry culture of human tubercle bacilli, and a month later 1 centigramme, are refractory to bovine tuberculosis Thomassen, in order to reach the same result, followed a different procedure.

The experiments of Thomassen have proved that calves can be infected by injecting large doses of human tubercle bacilli into the veins, especially if the animal possesses a diminished resistance to tuberculosis, and that the pathogenic properties of this virus is modified according to the origin and by prolonged artificial culture; they have also shown that inoculation with human tuberculosis in young bovines produces in about fifteen days dyspnœa, cough, and a thermic reaction, more or less marked, which may be transient or continue for several weeks.

The author endeavored to produce immunity to bovine tuberculosis by injecting human bacilli. The experiments included five calves. One was inoculated into the eye and two into the veins with fresh cultures obtained in the former case from the kidney of a man, and in the latter from expectoration cultivated on potato after passage through a guinea-pig. After disappearance of the thermic reaction, one of the calves inoculated into the veins and the one infected through the eye were tested with tuberculin. The first gave no reaction; the second one of 1.1°. The three calves were then inoculated once or several times with fresh cultures of bovine bacilli. Each inoculation was followed in twenty-four hours by a thermic reaction. Eight or nine months after the beginning of the experiment the calves were killed. Two control calves were infected with bovine cultures; one inoculated into the jugular vein with 30 milligrammes died in thirty days; the other, which received successively 8 and 30 mm., was destroyed two months afterward.

From the lesions found at the autopsy, Thomassen made the following conclusions: Bovida support quite easily large intravenous injections of human bacilli (30 milligrammes); inoculation with a small quantity confers a certain degree of immunity to bovine tuberculosis. Fresh cultures from expectorations possess less virulence than those obtained from internal organs or the milk. The thermic reaction consecutive to the first injection occurs in from ten to fifteen days; after the second and third, in twenty-four hours. It is more marked and more prolonged after inoculation with bacilli than after an injection of tuberculin.—*Rec. de Med. Vet.*

STRYCHNINE INTOXICATION. Danou employed intrapleural injections of sulphate of strychnine, in 5 c.c doses, of a saturated solution, to destroy dogs, whether healthy or diseased. Death follows very rapidly when the nervous system is normal. It is more tardy even when the strychnine is given in larger doses if the nervous system is affected with a disease. This result was seen in the following five cases: Acute meningitis, tubercular meningitis, chorea, Parkinson's disease, and paralysis.—*Rev. Vet.*

THE ROLE OF CHOLIN IN EPILEPSY. By means of the lumbar puncture of Quincke, employed in man for the diagnosis and treatment of nerve diseases, the cerebrospinal fluid can be collected for chemical analysis. In 15 out of 18 cases of epilepsy this liquid contained cholin. Solutions of pure cholin injected into the cranial cavity of rabbits produced tonic and clonic contractions, often accompanied by paralysis, dyspnœa, salivation, peristalsis with frequent evacuations of feces and urine, and dilatation followed by contraction of the pupils. Control animals in which a salt solution was injected showed no symptoms, excepting, in some cases, muscular tremblings of different regions.

Donath justifies the conclusion that cholin acts as a poison upon the nervous system and plays an important role in epileptic attacks.—*Orvosi Hetilop.*

PARASITIC DERMATITIS. A Belgian horse, aged 8 years, presented every summer for three years an extremely tenacious dermatitis. It was localized at the head and wherever the harness and the shafts rubbed the skin. The skin showed bald patches covered with papules, from which escaped a small, caseous granule with a calcified centre. A pruriginous excoriated wound subsequently formed, which cicatrized slowly. Microscopic examination of the contents of the papule revealed the presence of the larva of a nematode discovered by Rivalta.—*Rev. Vet.*

NEURECTOMY FOR SPAVIN. Bossi has employed the operation of Bossi five times in cases that resisted firing. The horses were so lame that they were about to be sent to the abattoir. The wound from resection of the sciatic cicatrized four times by first intention; that from tibial neurectomy always suppurated. Two cases were at work two and one-half years after the operation; one lost the hoof in six months; two could not be traced. Double neurectomy is indicated for spavins that resist ordinary treatment.—*Il moderno Zooiatro.*

REMARKABLE CASE OF LONGEVITY. Meynard gives the following information on positive authenticity. A mare, "Dulcinée," belonging to the Marquis of Ferronnays, was hunted in 1864. Supposing that she was, at most, only four years old at this time, she should now be forty-three years old. The mare is still living and in good health, and in 1896, at the age of thirty-six years, gave birth to a foal.—*Rev. Gen. de Med. Vet.*

Senile Lesions of the Nervous System. Vallée, of Alfort, investigated the senile lesions of the cerebrospinal ganglia and their analogy with the neuronophagia seen in rabies. He examined microscopically the ganglia of thirty old dogs, varying in age from eight to twenty-one years, sacrificed as incurable or senile. All were free from rabies. In most of these old dogs about one-third or one-fourth of the capsules of the ganglia in section showed a peculiar aspect. Certain nerve cells were completely destroyed. The nerve cell surrounded by the endothelial capsule was replaced by leucocytes ; in other points the nerve cell, still visible, had lost its chromotophilic elements, and was surrounded by a crown of phagocytes. The ganglionic stroma itself was infiltrated with numerous leucocytes, which, at certain points, were agglomerated into considerable masses. Neuronophagia is, therefore, evident—that is, a normal phagocytosis of the nerve elements in aged animals. No pre-existing condition was recognized in the animals capable of explaining this change.

These observations give additional proof of the doctrine of Metchnikoff as to the role of leucocytes in atrophic changes. The author also claims that this senile lesion of the ganglia may be mistaken for the ganglionic alterations in rabic animals prematurely killed. In old dogs, therefore, he considers the ganglionic lesions, in the absence of other signs, insufficient to make a diagnosis.—*Revue Generale de Med. Vet.*

Arsenic as a Normal Constituent of the Body. Bertrand has made researches upon this question, and in quantities less than a milligramme has found this metal in several animal species—hairs, hoofs of bovines, and hairs and claws of the dog. He has also found it in animals other than mammals removed from all possible contamination by the industries, such as fishes, cetaceans, sponges, and other marine animals. Bertrand concludes that, like carbon, nitrogen, sulphur, and phosphorus, arsenic is one of the fundamental constituents of protoplasm. This discovery opens a new field for chemists and physiologists, and has a toxicological bearing in expert medicolegal investigations.—*Ann. de l'Institute Pasteur.*

Treatment of Knuckling. Cailliband reports a Percheron mare, twelve years old, lame for eighteen months from knuckling in the third degree in the right fore-leg. There was contraction of the tendons and exostoses of the pastern, deforming the hoof.

High double plantar neurectomy was performed and then tenotomy of the perforans and perforatus. The position of the fetlock remained the same and the lameness persisted. Subsequently median neurectomy with mechanical traction permitted the fetlock to reach its normal angle. The author concludes that treatment of knuckling should be attempted in this manner.

[The above operation may become complicated by suppuration of the foot, and better success has been obtained by substituting median neurectomy for high plantar resection.]—*Rev. Vet.*

CHOREA OF THE DIAPHRAGM. Chorea of the diaphragm, clonic spasm of the diaphragm, hiccough, thumps, etc., quite frequent in the horse, may result from the ingestion of cold water (Goubaux), but results most frequently from fatigue. Most authors, among them Thomassen, basing their opinion upon the experimental results of Schiff, contend that the spasms and the cardiac pulsations are isorhythmic.

Dupas relates a case clearly contradicting this view. The attack occurred suddenly after two days of severe army manœuvres. The spasms were regular as to force and rhythm, numbering 86 per minute, very violent, shaking the entire body, especially the thorax. The shock was especially palpable along the cartilages of the asternal ribs, the attachment of the diaphragmatic muscle. There was no contraction of the abdominal muscles. The heart could not be auscultated, but palpation with the hand showed it to beat from 72 to 75 per minute; pulse small and irregular. These symptoms gradually diminished, and in four days the animal had recovered. On the second day the pulse was 50 and the diaphragmatic contraction 60; on the third day these were, respectively, 40 and 25.

Three principal points are called attention to in this observation:

1. The frequency of the spasms, 86 per minute, at the beginning.
2. The changing numerical relation between the spasms and the cardiac pulsations during the course of the disease.
3. The regular rhythm of the spasms contrasting with the irregularity and the intermittence of the pulse.

From the last two conditions the author cannot substantiate the hypothesis of Thomassen, Haubner, and Siedamgrotzky, of the direct excitation of the phrenic nerves by the cardiac pulsations. Physiology teaches that in all fatigued muscle tissue there is a formation of lactic acid whose action upon the nerves provokes contractions; and this principle applies to a fatigued diaphragm. The contractions are comparable to the convulsive spasms of the members seen in the deer and hare chased for long distances.—*Rev. Gen. de Med. Vet.*

